# A KLK4 proteinase substrate capture approach to antagonize PAR1

**DOI:** 10.1038/s41598-021-95666-4

**Published:** 2021-08-09

**Authors:** Eitan Rabinovitch, Koishiro Mihara, Amiram Sananes, Marianna Zaretsky, Michael Heyne, Julia Shifman, Amir Aharoni, Morley D. Hollenberg, Niv Papo

**Affiliations:** 1 Avram and Stella Goldstein-Goren Department of Biotechnology Engineering, National Institute of Biotechnology in the Negev, Ben-Gurion University of the Negev, P.O.B. 653, 84105 Beer-Sheva, Israel; 2 Department of Physiology and Pharmacology, Cumming School of Medicine, University of Calgary, Calgary, Canada; 3 Department of Life Sciences, National Institute of Biotechnology in the Negev, Ben-Gurion University of the Negev, Beer-Sheva, Israel; 4 Department of Biological Chemistry, The Hebrew University of Jerusalem, Givat Ram Campus, 91906 Jerusalem, Israel

**Keywords:** Biophysical chemistry, Proteases, Enzymes, Proteases, Molecular engineering, Protein design, Drug development

## Abstract

Proteinase-activated receptor-1 (PAR1), triggered by thrombin and other serine proteinases such as tissue kallikrein-4 (KLK4), is a key driver of inflammation, tumor invasiveness and tumor metastasis. The PAR1 transmembrane G-protein-coupled receptor therefore represents an attractive target for therapeutic inhibitors. We thus used a computational design to develop a new PAR1 antagonist, namely, a catalytically inactive human KLK4 that acts as a proteinase substrate-capture reagent, preventing receptor cleavage (and hence activation) by binding to and occluding the extracellular R41-S42 canonical PAR1 proteolytic activation site. On the basis of in silico site-saturation mutagenesis, we then generated KLK4_S207A,L185D_, a first-of-a-kind ‘decoy’ PAR1 inhibitor, by mutating the S207A and L185D residues in wild-type KLK4, which strongly binds to PAR1. KLK4_S207A,L185D_ markedly inhibited PAR1 cleavage, and PAR1-mediated MAPK/ERK activation as well as the migration and invasiveness of melanoma cells. This ‘substrate-capturing’ KLK4 variant, engineered to bind to PAR1, illustrates proof of principle for the utility of a KLK4 ‘proteinase substrate capture’ approach to regulate proteinase-mediated PAR1 signaling.

## Introduction

Activation of the G-protein-coupled receptor (GPCR) family of proteinase-activated receptors (PARs) by certain extracellular serine proteinases is known to trigger cell signaling^1^. The activation of PARs is initiated by enzymatic cleavage of their extracellular N-terminal sequence to expose a ‘tethered ligand’ that binds to sites on the extracellular PAR domains to trigger PAR-mediated cell signaling. The first potent activator of PAR1 to be identified and characterized was thrombin, a serine protease that cleaves PAR1 at its ‘canonical’ extracellular cleavage site between residues R41 and S42^2^. It was subsequently found that members of the kallikrein-related peptidase (KLK) family of serine proteinases are also able to cleave/activate PARs^3^. KLK4, KLK5, KLK6 and KLK13 cleave PAR1 at the canonical R41-S42 site, and KLK13 also cleaves PAR1 at position R46-N47^4^. A variety of other proteinases were found to regulate PAR1 activation by cleaving bonds within the A33 to E57 N-terminal sequence of human PAR1 at distinct sites both upstream and downstream of the ‘tethered ligand’ sequence^5^. These PAR-regulating proteinases include the matrix metalloproteinases MMP1, MMP2 and MMP13. Of note, the coagulation proteinase, activated protein C (APC), cleaves PAR1 at its R46/N47 site to unmask a tethered ligand sequence, thereby conferring biased signaling that differs from the activation of PAR1 by thrombin^6^. Thus, the PAR1 N-terminal sequence ^33^ATNATLDPRSFLLRNPNDKYEPFWE^57^ constitutes a common target in the sequence of PAR1 for activation by a variety of proteinases.

Following activation by extracellular proteinases, including members of the KLK family, PAR1 plays significant roles in many normal physiological processes, such as coagulation^7^, inflammation^8^, and vascular homeostasis^9^, but it also contributes to some inflammatory^10^ and cardiovascular pathologies, including atherosclerosis^11^, restenosis^12^, and thrombosis^13^, and to the progression of some cancers by stimulating cell migration and tissue invasion. Indeed, there is a strong correlation between PAR1 expression and the progression of lung^14^, prostate^15^, gastric^5^ and ovarian^16^ cancers and of melanoma^17^. In lung, ovarian and breast cancers and in melanoma, the activation of PAR1 promotes invasiveness and tumorigenicity^18–21^. For example, Boire et al. showed that MMP1 activation of PAR1 promoted invasiveness and tumorigenesis of breast cancer cells in xenograft models^19^; Villares et al. showed that in-vivo treatment of melanoma-bearing mice with PAR1 siRNA inhibited melanoma growth and metastasis^22^; and Adams et al. showed that PAR1 expressed by tumor stromal cells promoted colon cancer growth in vivo^22,23^.

The KLK family of serine proteinases represents the largest cluster of serine proteinases in the human genome. Expressed in a wide variety of tissues, the kallikrein-related peptidases are implicated in numerous pathophysiologies, ranging from cancers to skin disorders, such as Netherton syndrome^24–26^. Although the KLKs serve as attractive therapeutic targets in the management of certain inflammatory diseases and cancers, the development of selective KLK-targeted enzyme inhibitors represents a challenge^27^, due to the structural similarity, especially at the catalytic site, of KLK family members. In light of the potential role played by KLK4 (having a sequence homology of 31.64% with thrombin) in prostate cancer via activating either PAR1 or PAR2^28^, we sought to develop a strategy, alternative to generating a KLK4-specific enzyme inhibitor, to prevent KLK4-stimulated activation of PAR1. We thus aimed to prevent the ability of KLK4 to cleave/activate PAR1 by designing a high-affinity catalytically inactive KLK4 that could act as a ‘decoy’ substrate-capturing reagent by binding to PAR1 and thereby masking the activation site and blocking PAR1 activation by the proteolytically active wild-type KLK4.

Below we describe our novel approach for selectively blocking PAR1 activation by KLK4 and, by extension, other proteinases like the KLKs that might be present in the microenvironment of tumors^29^ and in inflammatory tissues. Our strategy is derived from the protease ‘substrate-capture or trapping’ approach, as recently reviewed^30^. This strategy uses catalytically inactive proteinases that bind to, but do not cleave, a substrate. Our approach to inhibitor design comprised engineering an inactive mutant of KLK4 to bind to PAR1 with higher than natural affinity, thus converting it from a PAR1 agonist to a PAR1 antagonist. Since proteases, such as KLK4, are difficult to engineer by applying directed evolution approaches, we utilized a purely computational method for KLK4 engineering before preparing the mutant proteins in the laboratory. Using a computational design, we generated a first-of-a-kind PAR1 inhibitor by mutating the S207A and L185D residues in wild-type KLK4, a natural activator of PAR1 that strongly binds to PAR1. The strong binding of the mutant protein, designated KLK4_S207A,L185D_, to the extracellular N-terminal region of PAR1 blocked the canonical cleavage site of PAR1, thereby preventing the activation of PAR1 by KLK4. To demonstrate the potential of our newly designed inhibitor of PAR1-activating proteinases present in the tumor microenvironment, we investigated its ability to inhibit PAR1 activation in PAR1-overexpressing cancer cells, and to block the migration and invasion of human melanoma cells lines that endogenously expresses PAR1 ^17^, namely, WM3682 and the highly invasive line WM3314. Our results provide an evaluation of the potential of using the proteinase substrate-capture strategy to block the cleavage/activation of a selected proteinase target, namely, PAR1.

## Results

### Predicting mutations in KLK4 that increase its affinity for the PAR1 peptide

**Figure 1:**
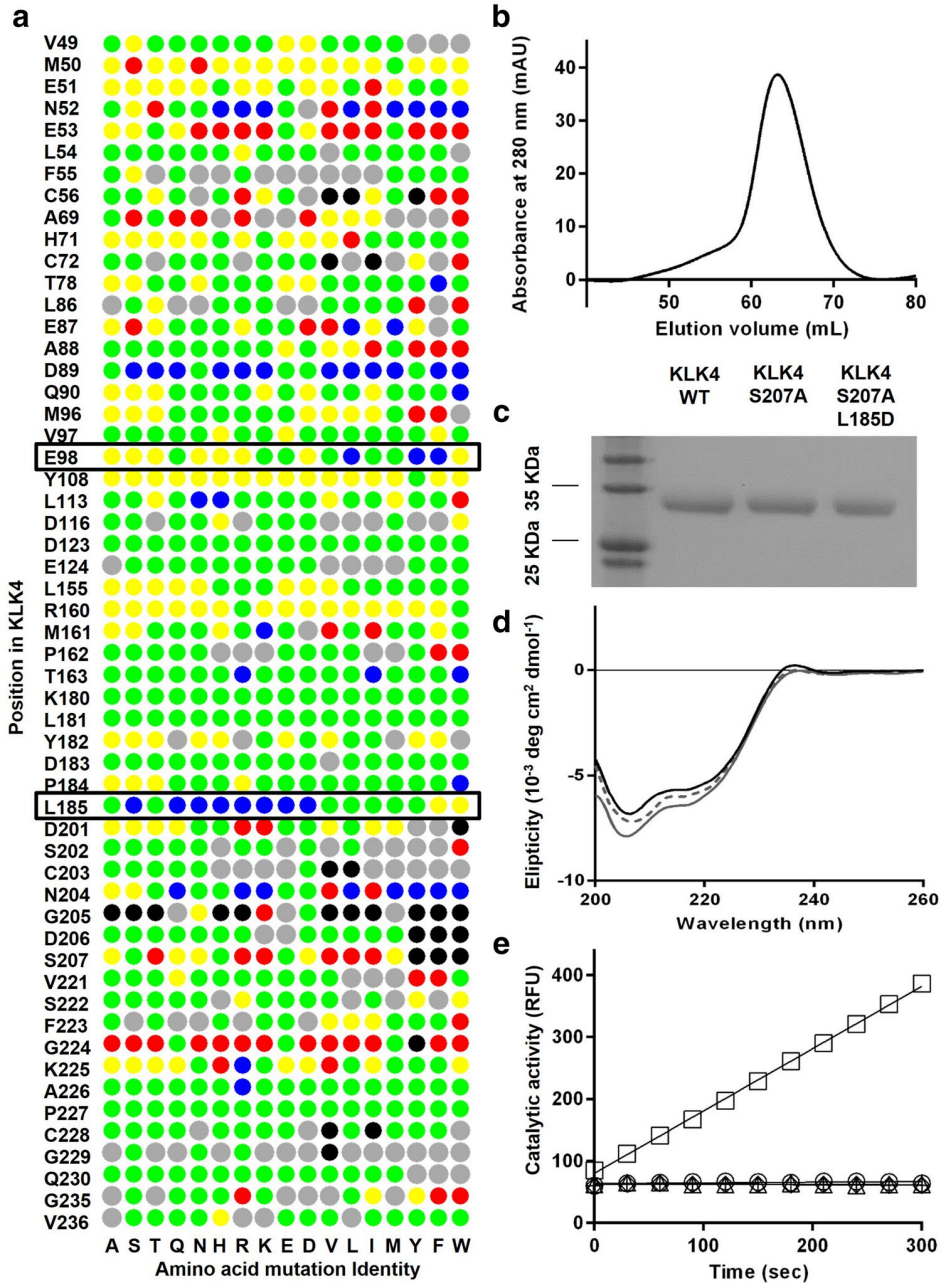
Affinity predictions, purification, and characterization of KLK4 variants. (**a**) The computational binding landscape of KLK4/PAR1 interactions. KLK4 binding interface residues and their identity in the wild-type sequence are shown on the left, and the amino acids to which they were mutated are shown on the bottom. The change in binding free energies conferred by the mutations are color coded according to the following characteristics: blue—stabilizing, green—neutral, yellow—slightly destabilizing, and red—strongly destabilizing. The gray dots represent mutations that destabilize KLK4_S207A_. The calculations were performed using the in silico saturation mutagenesis protocol of Sharabi et al. (**b**) A representative size-exclusion chromatography (SEC) UV absorbance signal of pro-KLK4 proteins (shown here for KLK4_WT_) after the Ni–NTA elution purification step. (**c**) SDS-PAGE of KLK4_WT_ and of KLK4_S207A_ and KLK4_S207A,L185D_ as selected purified pro-KLK4 variants. (**d**) CD spectra of KLK4_WT_, KLK4_S207A_ and KLK4_S207A,L185D_ (10 µM), as indicated with a solid black line, a dashed line and a solid gray line, respectively. (**e**) Catalytic activity of KLK4 _WT_ and the KLK4 variants KLK4_S207A_ and KLK4_S207A,L185D_. A BOC-VPR-AMC fluorescent substrate was added to KLK4_WT_ (□), KLK4_S207A_ (Δ), and KLK4_S207A,L185D_ (○), all at final a concentration of 625 nM, or to buffer (control, ◊), and the fluorescent signal upon substrate cleavage was measured.

The in-silico saturation mutagenesis approach previously developed in our group allows us to predict affinity-enhancing mutations in any protein–protein interaction by starting from a high-resolution structure of the protein complex. Since the structure of the KLK4/PAR1 complex as such is not available, we modeled the structure of this complex by using the structure of KLK4 and the structure of thrombin (which is homologous to KLK4) interacting with PAR1 (see Methods). Using this modeled structure of the KLK4/PAR1 complex, we identified the binding interface of KLK4, namely, the 14 positions that interact directly with the PAR1 peptide. At these 14 positions, we introduced all possible mutations, one at a time, and computed the change in free energy of binding due to each mutation (Fig. 1a). This step enabled us to predict a number of affinity-enhancing mutations at several KLK4 positions (Fig. 1a). We then visually inspected each predicted affinity-enhancing mutation in the modeled KLK4/PAR1 structure, since experimentally we could only assay a small number of mutants. Our inspection showed that the most promising mutations were those at positions 89, 204 and, particularly, 98 and 185, for which strongly affinity-increasing mutations were predicted.

### Expression, purification and characterization of KLK4 mutants

The KLK4 variants selected for expression and purification all included the mutation S207A (or Ser195 according to chymotrypsin numbering; a member of the catalytic triad, which was previously reported to abolish the catalytic activity of KLK4^31^) and an additional mutation at either position E98 or position L185 (which both showed the highest potential for affinity increase by our calculations). The double KLK4 mutants tested were thus those with the S207A mutation in addition to one of the following mutations: E98I, E98Y, E98F, L185S, L185Q, L185N, L185H, L185R, L185K, L185E, or L185D. The active protease, KLK4_WT_, and the inactive low-affinity single mutant, KLK4_S207A_, were also purified and used as controls.

To test which of the catalytically inactive KLK4 mutants exhibited increased affinity to the PAR1 peptide, the mutants were expressed and purified as soluble proteins using the *Pichia pastoris* yeast strain and the pPic9K expression vector, a methodology that allows the purification of the recombinant proteins from the growth medium. All variants were expressed as zymogens (inactive enzyme forms in which a pro-peptide, with the sequence S^27^CSQ^30^, is fused to the N-terminus of the enzyme) tagged with a FLAG tag on the N-terminus and a 6 × His tag on the C-terminus. Activation of the enzyme was achieved with thermolysin by cleavage of the bond between the pro-peptide and peptidase domain. Western blot analysis verified the expression of all KLK4 variants except for KLK4_S207A,L185S,_ KLK4_S207A,L185N,_ KLK4_S207A,L185R_ and KLK4_S207A,L185K_ (data not shown), and these mutants were therefore not included in the subsequent experiments. The remainder of the KLK4 protein variants were purified using Ni–NTA affinity column, analyzed by mass spectrometry (Fig. S1), and activated with thermolysin to remove the N-terminus pro-domain, followed by size-exclusion chromatography (Fig. 1b,c, Fig. S1).

Before proceeding with the subsequent experiments, it was necessary to confirm that the secondary structure of KLK4 had not been altered upon generation of the S207A and L185D point mutations. To this end, we performed a circular dichroism (CD) analysis of pro-KLK4_WT_, pro-KLK4_S207A_ and pro-KLK4_S207A,L185D_. The CD spectra of the two mutants were very similar to the spectrum of pro-KLK4_WT_ (Fig. 1d), indicating that the secondary structure had remained intact.

### The L185D mutation increases the affinity of KLK4_S207A_ to the PAR1 peptide in vitro

**Figure 2:**
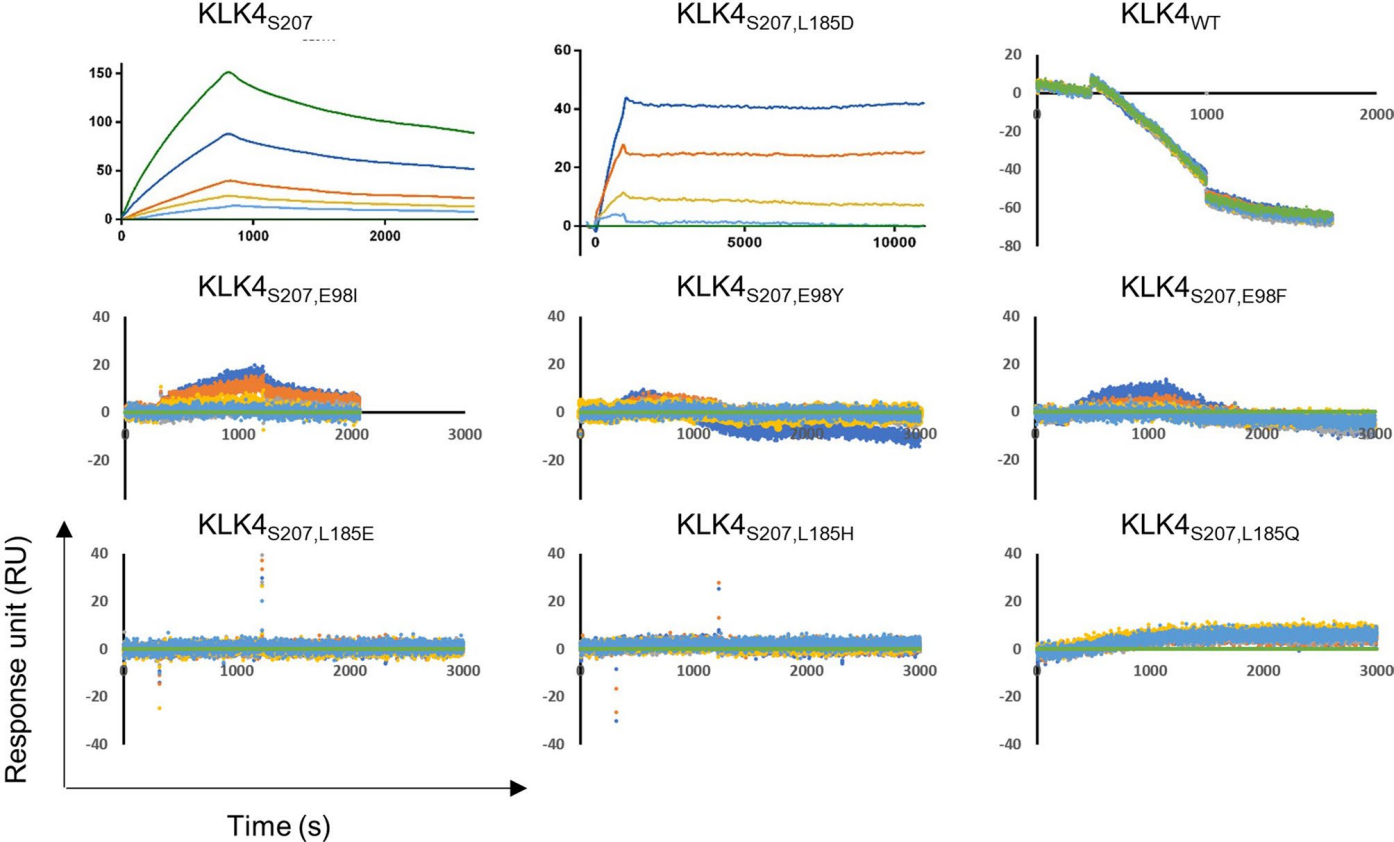
SPR sensorgrams of the binding interactions between a biotinylated PAR1 peptide, immobilized on a ProteOn NLC chip, and soluble KLK4_S207A_ (0.125–2 µM), KLK4_S207A,L185D_ (0.5–8 µM), KLK4_WT_ (6.25–100 nM), KLK4 _S207A,E98I/Y/F_ (0.125–2 µM) and KLK4_S207A,L185E/H/Q_ (0.125–2 µM). The analyses of the association and dissociation of KLK4 variants upon addition to the chip-immobilized PAR1 were carried out for 818 s and for 30 min, respectively, except for KLK4_S207AL185D_, for which the dissociation step was extended to 166 min.

To test whether the S207A mutation did indeed abolish the catalytic activity of all the KLK4 mutants tested, the catalytic activity of each mutant (vs. that of KLK4_WT_) was determined by measuring the fluorescent signal generated when reacting it with the Boc-VPR-AMC fluorescent substrate (Fig. 1e). In addition, binding of these proteins to an immobilized PAR1 peptide was determined by SPR (Fig. 2). As expected, only KLK4_WT_ was able to cleave Boc-VPR-AMC, while the S207A mutation abolished the catalytic activity of all the other KLK4 variants. In addition, the SPR results showed that KLK4_WT_ did not bind to the PAR1 peptide, but instead cleaved it rapidly. KLK4_S207A_, the catalytically inactive low-affinity mutant, having a single residue mutated in its catalytic pocket, showed no catalytic activity and bound the PAR1 peptide with a K_D_ of 1.12 × 10^−6^ M, which was calculated from the affinity rate constants K_on_ (3.49 × 10^2^ M^-1^ s^-1^) and K_off_ (3.91 × 10^–4^ s^-1^). The addition of an affinity-enhancing mutation, such as L185D (giving the KLK4_S207A,L185D_ double mutant), increased the affinity of the soluble protein to the PAR1 peptide by 20-fold (K_D_ = 5.55 × 10^–8^ M). The affinity enhancement resulted from an improvement in the dissociation rate (K_off_ = 3.17 × 10^–6^ s^-1^, which was 123-fold slower than that for the parental KLK4_S207A_), despite the slower association rate (K_on_ = 5.7 × 10 M^-1^ s^-1^) (Fig. 2). Thus, the incorporation of the L185D mutation induced stronger and tighter binding to the PAR1 peptide. All the other purified KLK4 double mutants showed no improvement in affinity toward the PAR1 peptide in comparison with KLK4_S207A_ (Fig. 2).

In summary, mutation S207A abolished the catalytic activity of KLK4, and engineering of the mutation L185D into the inactive KLK4_S207A_ variant increased its affinity to the PAR1 peptide by 20-fold. Therefore, in all subsequent experiments, it was the KLK4_S207A,L185D_ double mutant that was investigated as a functional antagonist of the PAR1 peptide.

### KLK4_S207A,L185D_ inhibits cell-expressed PAR1 cleavage

**Figure 3:**
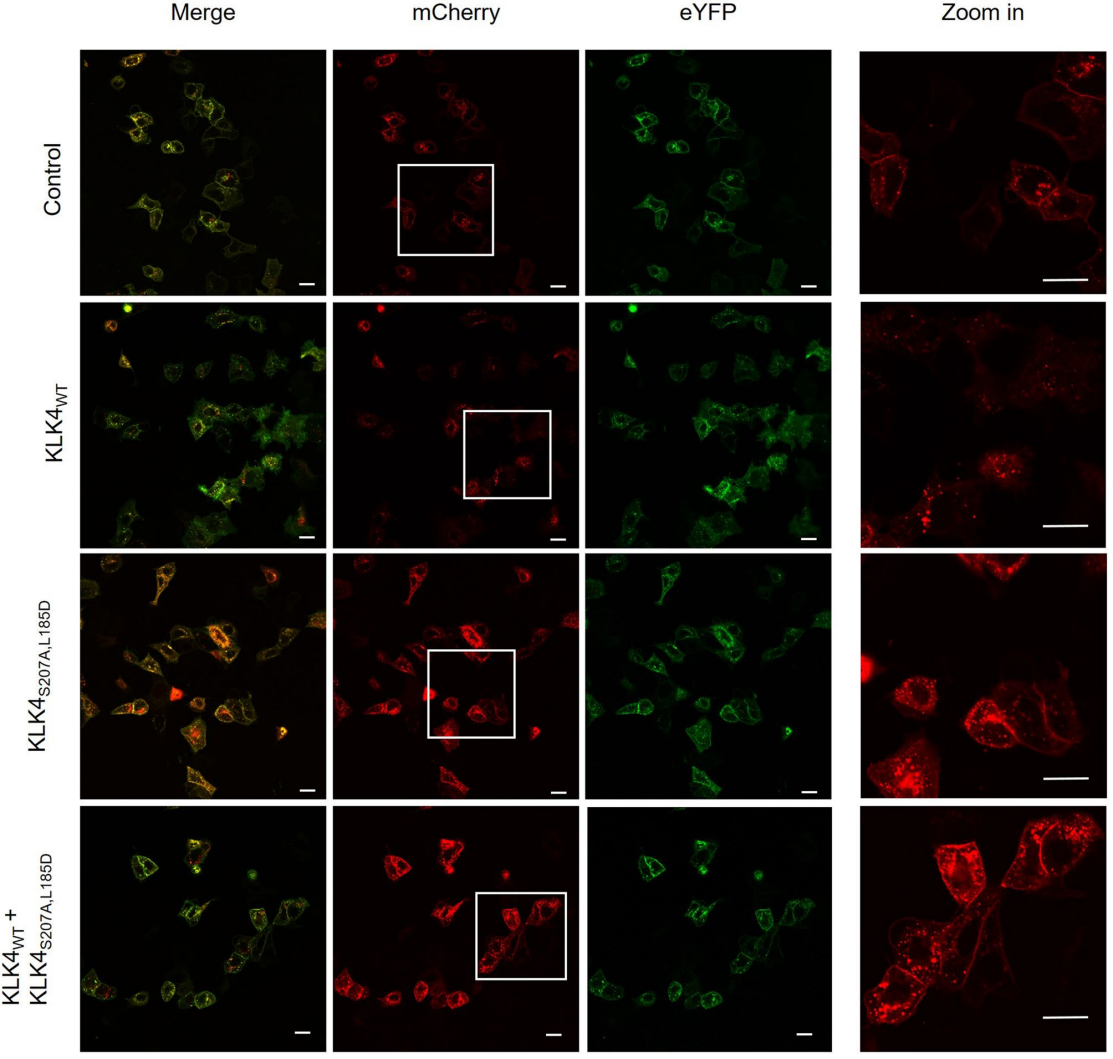
Confocal microscope images of MCF7 breast cancer cells, expressing the mCherry-PAR1-eYFP construct in the presence or absence of KLK4_WT_ and KLK4_L185D,S207A_. The right-most column represents a zoom-in view of the white box for each row in the mCherry column and arrows point to a visible membrane mCherry (extracellular PAR1 domain) signal. The red and green channels were used to measure mCherry (extracellular PAR1 domain) and eYFP (intracellular PAR1 domain), respectively. A 20-µm scale bar is indicated as a white bar on the lower right corner of each image.

To test whether KLK4_S207A,L185D_ inhibits cleavage of the full-length wild-type PAR1 transmembrane receptor by KLK4_WT_ (i.e., whether KLK4_S207A,L185D_ serves as a potential PAR1 antagonist) in an in-vitro cellular system, we used the breast cancer MCF7 and melanoma WM3682 cell lines as model systems. The full-length PAR1 protein, fused to an mCherry fluorescent protein at the N-terminus and an eYFP fluorescent protein at the C-terminus, was overexpressed in these cells. As in the wild-type PAR1 protein, the N-terminus (which houses the cleavage site for the KLK4_WT_ peptidase) of the fluorescent fusion PAR1 protein is localized in the extracellular region, and the C-terminus is located in the intracellular region. In both the MCF7 and WM3682 cell lines, cleavage (activation) of PAR1 upon treatment with 100 nM KLK4_WT_ removed the extracellular mCherry, leaving the intracellular eYFP intact (Fig. 3 and Supplementary Fig. S2). As may be seen in the confocal microscopy experiments described here and as was also found in previous studies (e.g.^32^), the released mCherry tag appeared to be internalized into the cells. This finding stands in contrast to that for the untreated cells, for which the extracellular domain (mCherry) underwent only a minor cleavage, probably caused by endogenously released extracellular proteases. Notably, addition of 200 nM KLK4_S207A,L185D_ together with 100 nM of the catalytically active KLK4_WT_ inhibited the cleavage of membrane PAR1.

**Figure 4:**
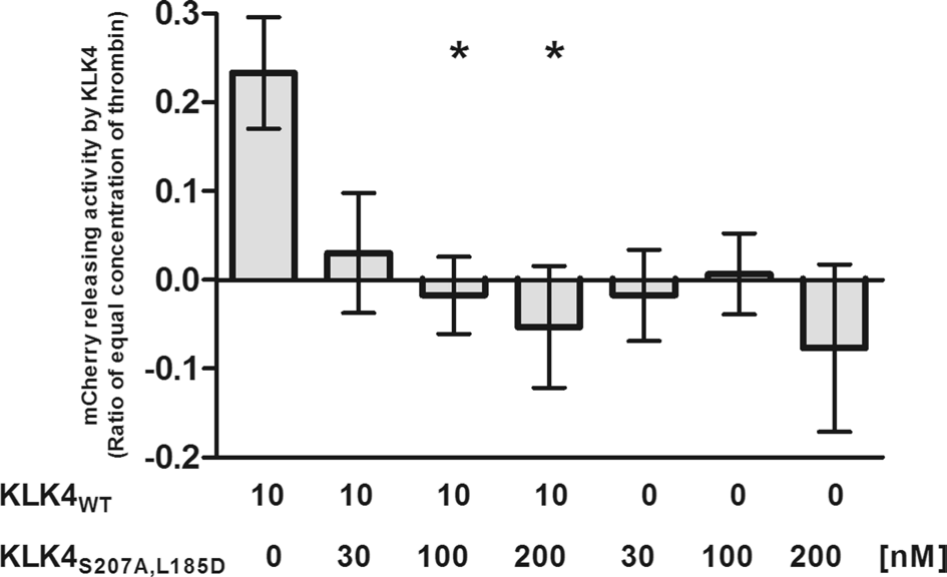
Inhibition by KLK4_S207A,L185D_ of the ability of KLK4_WT_ to cleave the N-terminus of PAR1. Fluorescence of mCherry released from the PAR1 N-terminus by KLK4_WT_ in the absence or presence of increasing concentrations of the substrate-capture KLK4_S207A,L185D_ mutant of KLK4 was detected in supernatant of mCherry-PAR1-YFP transfected WM3682 indicator cells, as outlined in Methods. The release of the N-terminal mCherry tag of PAR1 by wild-type KLK4 (1 U/mL) was expressed as a ratio of the release caused by 1 U/mL/10 nM thrombin in the same experiment. The KLK4-mediated cleavage-release of the PAR1 N-terminus was blocked by KLK4_S207A,L185D_, which alone did not release the N-terminus (right-hand histograms). Data showing the mCherry release caused by KLK4 relative to that caused by 1 U/mL thrombin represent the average fluorescence release ± SEM for 4 replicate monolayer samples. *, *P* < 0.05 by one-way ANOVA for the release in the presence, vs the absence, of KLK4_S207A,L185D_.

In a complementary, more quantitative, experiment using fluorescence spectroscopy, we showed that treatment of WM3682 cells overexpressing fluorescently labeled PAR1 with a mixture of KLK4_WT_ and KLK4_S207A,L185D_ resulted in a significant, dose-dependent inhibition of PAR1 cleavage in comparison with treatment with KLK4_WT_ alone (Fig. 4). Of particular importance, in the absence of KLK4_WT_, inhibition by KLK4_S207A,L185D_ of the PAR1 cleavage, presumably mediated by endogenously produced microenvironment proteinases, was also observed.

### KLK4_S207A,L185D_ inhibits melanoma cell migration

**Figure 5:**
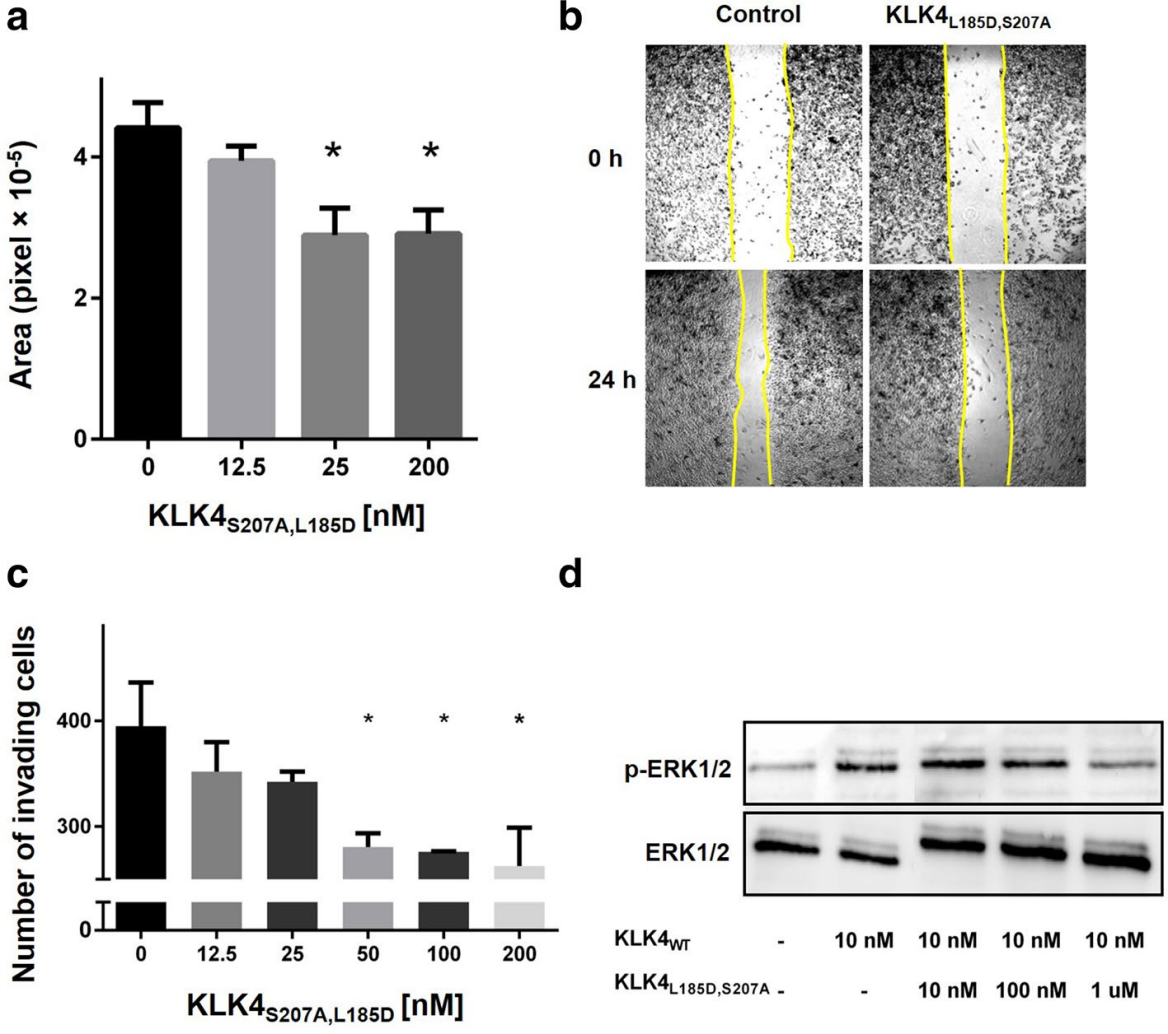
Inhibition of cell invasion, migration and downstream signaling of melanoma cells by KLK4_S207A,L185D_. (**a**) Inhibition of WM3682 melanoma cell migration as determined by a scratch assay. A monolayer of WM3682 cells was scratched linearly. After the scratch had been generated, the cell culture was supplemented with 0, 12.5, 25 and 200 nM KLK4_L185D,S207A,_ and the cells were then imaged by a phase-contrast microscopy, at 0 and 24 h after the scratch had been made. The relative area covered by cells for each treatment was calculated as the ratio of the scratch gap at 24 h and the original gap at 0 h. The results are presented as means ± SE (n = 3). *p < 0.05 compared with the untreated control by ANOVA. (**b**) Representative images of cells that had migrated (from panel **a**). (**c**) Inhibition of invasion of WM3314 melanoma cells by KLK4_L185D,S207A_ was determined in a Boyden chamber invasion assay. The numbers of cells that migrated through the membrane were counted at 18 h post treatment with 0, 12.5, 25, 50, 100 and 200 nM KLK4_L185D,S207A_. The results are presented as means ± SE (n = 3). *p < 0.05 compared with the untreated control by ANOVA. (**d**) Inhibition of ERK1/2 activation by KLK4_L185D,S207A_. WM3682 cells were treated for 15 min with PBS, 10 nM KLK4_WT_, or with a mixture of 10 nM KLK4_WT_ and 10 nM, 100 nM or 1 µM KLK4_L185D,S207A_, and analyzed by western blotting for ERK and p-ERK levels.

PAR1 activation has been shown to promote the motility of melanoma cells^33^. To test whether addition of KLK4_S207A,L185D_ could inhibit melanoma cell migration via inhibition of PAR1 activation, we performed a scratch-induced migration assay of melanoma WM3682 cells supplemented with different concentrations of KLK4_S207A,L185D_ (Fig. 5a and b). Prior to performing this assay, we confirmed that WM3682 cells do indeed express high levels of endogenous PAR1 (by using flow-cytometry; Supplementary Fig. S3a) and that KLK4_S207A,L185D_ does not alter cell proliferation in any way that could result in misinterpretation of the scratch assay results. For the latter purpose, we compared the proliferation after 24 h of incubation of WM3682 cells treated with 1 µM KLK4_S207A,L185D_ with that for untreated cells; the results showed no difference in cell proliferation (Supplementary Fig. S4a). In the scratch assay, addition of 12.5 nM KLK4_S207A,L185D_ to WM3682 cells, immediately after the cell scratch was generated, reduced the spontaneous migration of the cells by 11% after 24 h vs. the scratch area covered with untreated control cells. When the concentration of KLK4_S207A,L185D_ was doubled to 25 nM, WM3682 cell migration was reduced by 34%, namely, a similar result to that obtained with 200 nM KLK4_S207A,L185D,_ with no evidence of a reduction in cell viability.

### WM3314 melanoma cell invasion is inhibited by KLK4_S207A,L185D_

In addition to its contribution to cell motility, PAR1 activation has also been shown to promote metastasis of melanoma cells^21^. To test the ability of KLK4_S207A,L185D_ to inhibit WM3314 melanoma cell invasion, we used a Boyden chamber Matrigel invasion assay. Prior to performing this assay, we confirmed that WM3314 cells express high levels of endogenous PAR1 by using flow-cytometry (Supplementary Fig. S3b). Addition of 10% fetal bovine serum (FBS) to the medium in the lower compartment of the Boyden set-up resulted in cell invasion from the upper chamber through the Matrigel to the lower compartment. Treatment of the cells in the upper chamber with increasing concentrations of KLK4_S207A,L185D_ resulted in a dose-dependent inhibition of cell invasiveness (Fig. 5c). When comparing the number of invasive cells upon treatment with 200 nM KLK4_S207A,L185D_ with the number of untreated cells, we found that 33.5% fewer cells had become invasive upon treatment with KLK4_S207A,L185D_.

### KLK4_S207A,L185D_ inhibits KLK4_WT_-induced phosphorylation of MAPK/ERK

After confirming that KLK4_S207A,L185D_ had the ability to inhibit melanoma cell migration and invasion (Fig. 5a and c, respectively) by inhibiting PAR1 cleavage by KLK4_WT_ or other endogenous proteases (as shown by the confocal microscopy results in Fig. 3 and Supplementary Fig. S2), we examined the ability of KLK4_S207A,L185D_ to inhibit the activation of MAPK/ERK, which is a major downstream signal produced by PAR1 activation through the KLK4_WT_-induced cleavage of PAR1's extracellular region^17^ and an important downstream signaling effector involved in cell motility^34,35^. To this end, WM3682 melanoma cells were treated either with KLK4_WT_ alone to activate MAPK/ERK, or with a combination of KLK4_WT_ and increasing concentrations of KLK4_S207A,L185D_, or left untreated (Fig. 5d). The results showed that 10 nM KLK4 induced 61% enhancement in activation of MAPK/ERK in comparison with the untreated sample. Addition of 1 μM KLK4_S207A,L185D_ completely inhibited the phosphorylation of MAPK/ERK that had been induced by 10 nM KLK4_WT_, producing a signal that was equal to that of the untreated control cells (Fig. 5d and Supplementary Fig. S6).

## Discussion

Our findings establish proof of principle for the utility of the proteinase-capture approach to block the cleavage/activation of PAR1, a biologically important KLK4 targeted substrate. Although the proteinase-capture approach has previously been used to identify potential substrates by a ‘pulldown-proteomic’ approach, we believe our work is the first to demonstrate proof of principle for blocking the cleavage/activation of a proteinase substrate, such as PAR1. Using in-silico predictions for affinity-enhancing mutations and experimentally testing several selected double mutants that were based on the catalytically inactive KLK4_S207A_ mutant, we identified a high-affinity double mutant, KLK4_S207A,L185D_, that acts as a PAR1 inhibitor. The success of our computational prediction is noteworthy, since no high-resolution structure of the KLK4/PAR1 protein complex was available and modeling such structures tends to reduce the accuracy of predictions. Both catalytic inactivation and affinity enhancement of the KLK_S207,L185D_ mutant (expressed and purified in a soluble form), relative to KLK4_WT_, were validated using SPR measurements of this mutant in complex with the PAR1 peptide. We thus showed that while a ~ sixfold reduced *k*_*on*_ affinity rate constant was observed for the KLK_S207,L185D_/PAR1 peptide complex compared with that of the KLK_S207_/PAR1 peptide complex, the *k*_*off*_ affinity rate was 123-fold slower for the former, resulting in an 20-fold overall affinity enhancement for the KLK_S207,L185D_/PAR1 peptide complex.

The KLK4_S207A,L185D_ ‘decoy’ substrate-capture enzyme was able to block KLK4- mediated cleavage of PAR1 expressed in MCF7 and WM3682 cell lines (visualized by confocal microscopy and fluorescence spectroscopy). Further, treatment of the MCF7 and WM3682 cells overexpressing fluorescently labeled PAR1 with a mixture of KLK4_WT_ and KLK4_S207A,L185D_ resulted in a significant inhibition of PAR1 cleavage in comparison with treatment with KLK4_WT_ alone. Of added importance, inhibition of PAR1 cleavage, presumably caused by endogenously produced microenvironment proteinases, was also observed in the absence of added KLK4_WT_. This inhibitory effect resulted in reduction in migration induced by unknown PAR1-activating proteinases in the tumor cell microenvironment.

Several cell-secreted proteinases have been suggested as enzymes that can cleave/activate PAR1 in MCF7 cells and may thus serve as agonists of PAR1. MMP1, for example, which cleaves PAR1 at a non-canonical site, between residues D39-P40^24^, was found to be endogenously overexpressed in the MCF7 cell line^36^. Another member of the MMP family, namely, MMP-13, which activates PAR1 at a different non-canonical cleavage site, between residues S42-F43, was also shown to be expressed in MCF7 cells^37^. In addition, other members of the KLK family are known to be expressed in MCF7 breast cancer cells^38^, including: KLK5, which cleaves PAR1 at positions R41-S42 and F43-L44; KLK6, which cleaves PAR1 at positions R41-S42, S42-F43, F43-L44, L45-R46 and R46-N47; and KLK13, which cleaves PAR1 at positions R41-S42, F43-L44, and R46-N47^3^. It is now known that proteinases, such as MMP1, APC, and neutrophil elastase, can cleave PAR1 at ‘non-canonical’ sites to stimulate ‘biased signaling’^39^. To the best of our knowledge, no previous work has been performed to evaluate protease expression in WM3682 cells, but a screen of the Broad Institute Cancer Cell Line Encyclopedia (CCLE) mRNA expression database revealed that *MMP1* is highly expressed in most melanoma cell lines, suggesting that it could also be expressed in the WM3682 cell line and could therefore be involved in the cleavage process of PAR1 at a non-canonical cleavage site, between residues D39 and P40^26^. The KLK4_S207A,L185D_ ‘decoy’ could, in principle, block PAR1 activation at this site as well as at the ‘canonical’ activation site.

To investigate how KLK4_S207A,L185D_ interacts with the PAR1 extracellular region at the molecular level to block activation at both canonical and non-canonical cleavage sites, we examined the interaction of KLK4_S207A,L185D_ with the PAR1 extracellular region. Structural analysis of the modeled KLK4/PAR1 complex structure showed that the affinity enhancement in the KLK_S207A,L185D_/PAR1 complex (Supplementary Fig. S5a) is probably due the formation of a new hydrogen bond between the mutated L185D residue in KLK4 and residue N35 (a residue excluded from the canonical cleavage site) in the PAR1 peptide (Supplementary Fig. S5b). Moreover, as previously described, a negatively charged surface patch on KLK4 (between loop M50-E53 and loop D89-Q95) could serve as a recognition site for binding with the hirudin-like domain on PAR1 (residues 51–55, KYEPF) (Supplementary Fig. S5a), similar to the interaction of PAR1 with the anion-binding exosite of thrombin (exosite 1)^4,33,40,41^. In addition, we predicted that additional hydrogen bonds could be formed between residues F55, G215, S222 and G224 of the mutated catalytic pocket in KLK4_S207A,L185D_ and residues D39, R41 and S42 in PAR1 (Supplementary Fig. S5c). These hydrogen bonds could be formed in addition to the electrostatic interactions predicted between the positively charged R41 in PAR1 that becomes buried within the negatively charged surface cavity of KLK4 in the PAR1/KLK_S207A,L185D_ complex (Supplementary Fig. S5d); these hydrogen bonds might, in fact, be the same interactions that mediate the cleavage of PAR1 by KLK4_WT_. All the above-predicted interactions between KLK4_S207A,L185D_ and PAR1 would probably ‘hold’ PAR1 residues 35–55 in close proximity to KLK4_S207A,L185D_, thereby blocking the access of other proteases and preventing them from cleaving PAR1 anywhere between residues 35–55.

In summary, we have established proof of principle for converting a PAR1-activating proteinase into a high-affinity substrate-capture antagonist, using KLK4 as a prototype enzyme present in the microenvironment of many tumors. The use of our substrate capture approach has also expanded the understanding of the activation of PAR1 and its potential contribution to melanoma invasion. As an antagonist strategy, our approach could be used not only to target PAR1 activation in selected settings, but also to block the proteinase-mediated generation of active polypeptides from their precursors in the tumor microenvironment.

## Materials and methods

### Modeling the KLK4/PAR1 complex structure

The structure of the PAR1 extracellular peptide (^33^ATNATLDPRSFLLRNPNDKYEPFWE^57^) in complex with KLK4 was modeled on the basis of the available crystal structures of a thrombin/PAR1 complex (PDB-ID 3LU9)^42^ and the structure of the wild-type KLK4 (PDB-ID 4K8Y)^43^. Missing residues were added using the Modeller suit^44^. All water molecules and ions were removed. The crystal structure of KLK4 was aligned with that of thrombin (which is homologous to KLK4) in 3LU9, and the modeled PAR1 peptide was aligned with PAR1 of the same crystal structure. The resulting KLK4/PAR1 complex structure was optimized using FlexPepDock^45,46^, and the best ranked model according to the Rosetta generic full-atom energy score was used as the input to the in-silico saturation mutagenesis protocol.

### In-silico saturation mutagenesis of selected positions in the KLK4/PAR1 interface

The modeled structure of the KLK4/PAR1 complex was used as the input for all calculations, and hydrogen atoms were added to the structure by MolProbity^47^. The KLK4 residues within 4 Å of the PAR1 peptide in the modeled KLK4/PAR1 complex structure were identified as the binding interface positions (KLK4 positions 49–56, 69, 71–72, 78, 86–90, 96–98, 108, 113, 116, 123–124, 155, 160–163, 180–185, 201–207, 221–230, 235–236). For each of the above positions, we defined a set of shell positions that included the residues on KLK4 that are within 3 Å of the KLK4 position to be mutated and the residues on PAR1 that are within 5 Å of the KLK4 position. For each binding interface position, we then performed 17 calculations, in which the considered position on KLK4 was either kept as wild type or was replaced with any other amino acid, except proline, cysteine, or glycine. During the calculation, the shell and the interface residues were repacked, and the energies of the KLK4/PAR1 complexes were calculated for the wild-type and mutated complexes. The two proteins in each complex were then separated to enable calculation of the energy of each unbound chain separately without further repacking of the side chains. The intermolecular energy was calculated by subtracting the energies of the single chains from the total energy of the complex. ΔΔ*G*_bind_ was calculated as the intermolecular energy of the mutated complex minus the intermolecular energy of the wild-type complex. The obtained ΔΔ*G*_bind_ was normalized according to a linear equation obtained in our previous work in which the correlations between various experimental and computed ΔΔ*G*_bind_ values were determined^48^. Rotamer libraries used for computational modeling were based on the backbone-dependent library of Dunbrack and Karplus^49^, with additional rotamers expanded by 1 SD around their mean *χ*1 and *χ*2 values. For the calculations, we used ORBIT software with the energy function optimized for the design of protein–protein interactions^48^. The lowest-energy rotameric conformation of each mutated complex was found using the dead-end elimination theorem^50,51^.

### Recombinant expression and purification of KLK4 variants

KLK4_WT_ (amino acid sequence and numbering are according to UniProtKB—Q9Y5K2) and selected KLK4 variants (KLK4_S207A_, KLK4_S207A,E98I_, KLK4_S207A,E98Y_, KLK4_S207A,E98F_, KLK4_S207A,L185E_, KLK4_S207A,L185H_, KLK4_S207A,L185Q_ and KLK4_S207A,L185D_) were purified as previously described with minor modifications^52^. In brief, the pPIC9K plasmid (Invitrogen, CA, USA) containing the pro-KLK4_WT_ clone (with FLAG and His tags fused to the N- and C-termini of the protein, respectively) were isolated from individual *Escherichia coli* colonies by using a HiYield plasmid mini-kit (RBC Bioscience, Taiwan) and sequenced using primers 1 and 2 (Supplementary data Table S1). Point mutation S207A was introduced into the KLK4_WT_ clone by using site-directed mutagenesis PCR with overlapping primers 3 and 4 (Supplementary Table S1) and PrimeSTAR HS DNA polymerase (Takara Bio, Otsu, Shiga, Japan). The parental methylated plasmid in the reaction (pPICK9K plasmid containing KLK4_WT_) was digested with the DpnI enzyme, according to the product protocol (New England Biolabs, MA, USA). The final product (containing only the linear dsDNA product) was electroporated into *E. coli*, incubated at 37 °C with shaking at 300 rpm for 1 h in LB medium (1% yeast extract w/v, 2% peptone w/v, 2% dextrose w/v), plated onto LB-Amp plates (ampicillin at 1 mg/L), and then incubated at 37 °C for 24 h. Several colonies were scraped from the plate, transferred into LB-Amp medium, and incubated at 37 °C with shaking at 300 rpm for 24 h. The pPIC9K plasmid containing the KLK4_S207A_ clone was then isolated from each *E. coli* colony with a HiYield plasmid mini-kit (RBC Bioscience) and sequenced using primers 1 and 2 (see Supplementary Table S1). KLK4 point mutations E98I, E98Y, E98F, L185S, L185Q, L185N, L185H, L185R, L185K, L185E and L185D were separately generated in the pPIC9K plasmid containing the KLK4_S207A_ clone using the same procedure as that described above with primers 5–26, respectively (for example, E98I with primers 5 and 6; E98Y with primers 7 and 8 and so on; Supplementary Table S1). pPIC9K plasmids containing KLK4 mutants with the correct sequences were linearized using SacI, and ~ 30 µg of DNA was electroporated into *P. pastoris* strain GS115 (Invitrogen) for chromosomal incorporation by homologous recombination. Transformed cells were plated onto RDB plates (18.6% sorbitol w/v, 2% agar w/v, 2% dextrose w/v, 1.34% yeast nitrogen base w/v, 4 × 10^–5^% biotin w/v, 5 × 10^–3^% of l-glutamic acid, l-methionine, l-leucine, l-lysine, l-isoleucine w/v) and incubated for three days at 30 °C. Cells were scraped from the plates using E-buffer (0.12% Tris base w/v, 9.24% sucrose w/v and 0.02% MgCl_2_ w/v at pH 7.5) and plated onto YPD-G418 plates (4 mg/mL G418) for an additional three days. Colonies (~ 10) from each clone were harvested, seeded into 5 mL of BMGY medium (2% peptone w/v, 1% yeast extract w/v, 0.23% K_2_H(PO_4_) w/v, 1.1812% KH_2_(PO_4_) w/v, 1.34% yeast nitrogen base w/v, 4 × 10^–5^% biotin w/v, 1% glycerol v/v), and incubated at 30 °C with shaking at 300 rpm for 24 h. For protein expression, cells in BMGY medium were pelleted and then re-suspended in 5 mL of BMMY medium (2% peptone w/v, 1% yeast extract w/v, 0.23% K_2_H(PO_4_) w/v, 1.1812% KH_2_(PO_4_) w/v, 1.34% yeast nitrogen base w/v, 4 × 10^–5^% biotin w/v, 0.5% MeOH v/v) to reach OD_600_ of 1.0 and incubated at 30 °C with shaking at 300 rpm for 72 h, with 0.5% MeOH being added every 24 h. Protein expression and secretion into the medium were analyzed by western blot, using mouse anti-FLAG primary antibody (Sigma Aldrich, MO, USA) and an alkaline phosphatase conjugated anti-mouse secondary antibody (Jackson ImmunoResearch, PA, USA). BCIP reagent (Sigma Aldrich, MO, USA) was used for protein expression signal analysis according to the product protocol. Individual clones (representing each of the KLK4 variants) showing the strongest expression levels were selected and subjected to large-scale production and purification procedures. Briefly, clones in 5 mL of BMGY medium at OD_600_ of 8.0–10.0 were added to 500 mL of BMGY medium at 30 °C with shaking at 300 rpm for 24 h. Cells were precipitated from the medium and induced for protein secretion in 500 mL of BMMY at 30 °C with shaking at 300 rpm for 72 h. MeOH (0.5%) was added to the medium every 24 h. After induction, the supernatant was filtered through a 0.22-µm vacuum filter. The filtrate was then subjected to the following workup: NaCl was added to a final concentration of 300 mM, imidazole (Sigma, MO, USA) was added to give a final concentration of 10 mM, and the solution pH was adjusted to 8.0. After 1 h at 4 °C, the medium was filtered again, and the protein of interest was purified on a column packed with 5 mL of Ni–NTA Sepharose beads (Invitrogen). The protein was eluted from the Ni–NTA beads with 15 mL of buffer at pH 8.0 containing 50 mM sodium phosphate, 300 mM NaCl and 250 mM imidazole. The eluted protein was concentrated to 5 mL, and the buffer was replaced with PBS using a Vivaspin with a 5-kDa cutoff (Vivaproducts, MA, USA). The protein was then subjected to activation by thermolysin (Calbiochem, LaJolla, CA). Briefly, thermolysin, which cleaves the N'-terminus pro-domain, was added to KLK4 in a mass ratio of 1:80 (thermolysin:KLK4) for 2 h at 37 °C. The reaction was terminated by addition of 1,10-phenanthroline at a final concentration of 10 mM. Activated KLK4 proteins were then purified using gel filtration chromatography on a Superdex™ 75 16/600 GL column in an ÄKTA™ Pure (GE Biosciences, PA, USA) chromatography system. The gel filtration column was pre-equilibrated with PBS (pH 7.4), and the protein was eluted with PBS. The protein concentration was calculated from protein absorbance at 280 nm (extinction coefficient of 31,690 M^-1^ cm^-1^ and calculated mass of 26.4 kDa) obtained using a NanoDrop spectrophotometer (Thermo Scientific, MA, USA). Non-activated protein samples (not treated with thermolysin), with theoretical mass of 28.6 kDa, were subjected to SDS-PAGE (denatured and reduced) and mass spectrometry analysis (Ilse Katz Institute for Nanoscale Science Technology, BGU). The purified proteins were stored at −80 °C.

### Surface plasmon resonance (SPR) spectroscopy

The affinity constants for the interaction between the purified KLK4 variants and the PAR1 peptide were determined by SPR spectroscopy on a ProteOn XPR36 (Bio-Rad) instrument at the Cytometry, Proteomic and Microscopy Unit (NIBN, BGU). A biotinylated, PAR1 peptide (biotin-^31^SKATNATLDPRSFLLRNPNDKYEPFWED^58^-FITC, > 95%, Peptron, Daejeon, Korea; residue numbering based on UniProtKB-P25116, PDB-ID 3LU9) was immobilized on the surface of an NLC chip (NeutrAvidin immobilized to a GLC layer) via NeutrAvidin–biotin interactions. The PAR1 peptide (26 µg) dissolved in PBST (phosphate-buffered saline with 0.005% Tween 20) was immobilized on the chip to give 2600 response units (RU). An empty channel was used as the negative control. Prior to each binding assay, the temperature was set at 25 °C. Purified KLK4 variants were then allowed to flow over the surface-bound PAR1 peptide at concentrations of 100, 50, 25, 12.5, 6.25 and 0 nM for KLK4_WT_, concentrations of 2, 1, 0.5, 0.25, 0.125 and 0 µM for KLK4_S207A_, KLK4_S207A,E98I_, KLK4_S207A,E98Y_, KLK4_S207A,E98F_, KLK4_S207A,L185E_, KLK4_S207A,L185H_ and KLK4_S207A,L185Q_, and concentrations of 8, 4, 2, 1, 0.5 and 0 µM for KLK4_S207A,L185D_, at a flow rate of 30 µL/min for 818 s. The dissociation of the protein-peptide complex was then monitored with PBST flowing over the surface at a flow rate of 30 µL/min for 30 min except for KLK4_S207AL185D_, where this step was prolonged to 166 min (due to slow dissociation). For each protein complex, a sensorgram was generated from the RUs measured during the course of the interaction minus the values of the empty channel. The values of *k*_*on*_ and *k*_*off*_ were determined by fitting the sensorgrams to a 1:1 Langmuir kinetic model. The fitted data were considered statistically valid with a χ^2^ value that was less than the 10% of the RUmax in each of the fitted sensorgrams.

### CD spectroscopy

To examine whether any structural change had been caused as a result of the mutations to KLK4, we evaluated the secondary structure of the selected purified non-activated KLK4 proteins by CD spectroscopy (J-815 CD spectrometer, JASCO, Tokyo, Japan, with a 1-mm path length quartz cuvette). Spectra of 10 μM purified protein in 400 μL of PBS were obtained at 25 °C, and the signal of the blank solvent (PBS) was subtracted. Ellipticity (degree × cm^2^/dmol) was obtained from the normalized average of four spectra in the range of 200–260 nm. Data points with a diode voltage above 1000 V were excluded.

### Confocal microscopy

To follow the cleavage of full-length cell-expressed PAR1 by KLK4_WT_ and the inhibition of this cleavage by KLK4_S207A,L185D_, we used the WM3682 human melanoma cell line (generously provided by Carmit Levi, Tel-Aviv University) or the MCF7 human breast cancer cell line (generously provided by Isam Khalaila, BGU). The cells were transfected with a fluorescently labeled PAR1 plasmid (pCDNA3.1 containing the mCherry-PAR1-eYFP construct described previously^32^) and Lipofectamine™ LTX as a transfection reagent, according to product protocol (Thermo Scientific). For WM3682 cells, following 24 h of transfection, cells were sub-cultured in μ-slide 8-well ibiTreat microscopy chambers (ibidi, Martinsried, Germany) in DMEM containing 10% FBS and incubated for additional 24 h in a humidified incubator at 37 °C and 5% CO_2_. Cells were washed once with PBS, suspended in FBS-free DMEM, and incubated for another 24 h. Thereafter, the cells were treated with 100 nM KLK4_WT_ (positive control), with 200 nM KLK4_S207A,L185D_, with a combination of KLK4_WT_ and KLK4_S207A,L185D_, or with medium alone (negative control) for 1 h under the same conditions as those prior to the analysis. Live fluorescence images were acquired with a confocal laser-scanning microscope (ZEISS LSM880 confocal microscope) using the Plan Apochromat 63 × /1.4 oil DIC M27 objective (Ilse Katz Institute for Nanoscale Science and Technology Shared Resource Facility, BGU). The assay was performed in triplicate, and representative images are presented . For MCF7, the transfection was performed directly on the μ-slide 8-well ibiTreat microscopy chambers (ibidi) and after 24 h, the medium was replaced with FBS-free DMEM and the cells were incubated for another 1 h in a humidified incubator at 37 °C and 5% CO_2_. Thereafter, the cells were treated with 100 nM KLK4_WT_, 200 nM KLK4_S207A,L185D_, 200 nM KLK4_S207A_, a combination of KLK4_WT_ and KLK4_S207A,L185D_ or KLK4_WT_ and KLK4_S207A_, or with medium alone (negative control) for 1 h and incubated under the same conditions as those prior to the analysis. Cells were washed once with PBS, then fixed in 4% paraformaldehyde in PBS for 10 min at room temperature, washed again, and covered with PBS. Fluorescent samples were imaged using an FV1000 laser scanning confocal microscope (Olympus) with 488 and 561 nm solid state lasers and a × 40/1.25 NA silicon immersion objective (NIBN, BGU). Fluorescence images were acquired using the Flowview 10 ASW V2.1 software. The assay was performed in two replications (separate wells for each condition), and two fields from each well were examined.

### Monitoring the KLK4-mediated release of the N-terminal mCherry PAR1 tag

Two T25 flasks of confluent WM3682 cells were transfected with the mCherry-PAR1-YFP construct described previously^32^ and incubated for 24 h at 37 $^\circ$ C in a humidified incubator in an atmosphere of 5% CO_2_ in room air. Transfected cells grown to confluency in the two T25 flasks were then lifted with 0.25% trypsin in 1 mM-EDTA-supplemented PBS, pH 7.4. Cells were combined and subcultured in a 48-well multi-dish tray (Nunc 1 cm^2^ area) in 10% serum-supplemented DMEM and incubated for a further 24 h. Cells were serum starved by replacing the culture medium with 0.2 mL of serum-free DMEM containing 0.1% bovine serum albumin (BSA) and incubated overnight. The next day, cells were washed 3 times with isotonic Hanks’ balanced salt solution pH 7.4 containing 10 mM HEPES, 1.5 mM MgCl_2_, 1.5 mM CaCl_2_, and 0.1% BSA and replenished with 0.1 mL of the same buffer. KLK4_WT_ and KLK4_S207A,L185D_ were mixed and preincubated for 1 h on ice at 10 times the final specified concentration added to the cells. In each well, 10 µL of pre-incubated 10 × enzyme mix was then added to the indicator cell monolayer containing 90 µL of buffer and incubated for 30 min at 37 $^\circ$C. The supernatant was transferred to a 96-well multiwell plate and the intensity of fluorescence (excitation 560 nm/emission 620 nm) generated by the released mCherry tag was measured using SpectraMax i3X plate reader (Molecular Devices, San Jose, CA). The data show the average fluorescence ± SEM (bars) from 4 replicate wells relative to the signal generated by 1 U/mL thrombin (10 nM). The statistical difference for data obtained from the untreated vs. KLK4_S207A,L185D_ treated cells was determined by a one-way ANOVA for measurements performed for four cell monolayer replicates.

### PAR1 cell expression

The expression levels of PAR1 in WM3682 and WM3314 cells were determined using flow cytometry. In brief, 0.3 × 10^6^ cells were seeded in a 6-well plate in DMEM containing 10% FBS and incubated in a humidified incubator at 37 °C and 5% CO_2_ until reaching 80% confluence. The cells were then washed with a flow-cytometry buffer (0.1% BSA in PBS), harvested using a cell scraper, and incubated for 30 min in a flow-cytometry buffer or in a flow-cytometry buffer containing anti PAR1 Alexa Fluor 680 conjugated antibody (1 µg per 1 × 10^6^ cells, ATAP2 clone, Santa Cruz, CA, USA). Cells were washed once with a flow-cytometry buffer and analyzed using a flow cytometer. For the negative control, the same procedure was carried out with one exception: 0.25% trypsin solution was used to detach the cells from the 6-well plate (instead of a cell scraper). Analysis was carried out on an Accuri C6 Flow Cytometer (BD Biosciences, CA, USA), and data analysis was performed using a FlowJo software (Treestar, Inc., CA, USA).

### Migration assay

Testing the inhibition by KLK4_S207A,L185D_ of the migratory phenotype of the WM3682 cell line was performed by using a scratch assay, as previously described^53^. Briefly, WM3682 cells (1.5 × 10^5^) were cultured as confluent monolayers in a 24-well plate in DMEM containing 10% FBS and incubated in a humidified incubator at 37 °C and 5% CO_2_. When the cells reached 100% confluence, a scratch was formed by removing a strip of cells across the well with a p200 pipette tip. The scratched monolayers were then washed twice with PBS to remove non-adherent cells, and 500 µL of DMEM supplemented with 2% FBS, containing 12.5, 25, or 200 nM KLK4_S207A,L185D_ or PBS (control), was added. The wells were captured with an EVOS FL cell imaging system at × 4 magnification, both immediately after cell wounding (t_0_) and again after 24 h (t_24_). The experiment was performed in triplicate, and the images were analyzed using ImageJ software. The ratio between the gap areas at t_0_ and t_24_ was calculated.

### Invasion assay

An in-vitro Boyden chamber assay was performed as previously described with minor changes^53^. Briefly, ThinCert 24-well cell culture inserts (Greiner Bio-One, Germany) were coated with growth factor reduced Matrigel (Corning, NY, USA) diluted in DMEM (Biological Industries, Israel) at a 1:30 v/v ratio. The lower chamber was filled with 600 μL of DMEM supplemented with 10% FBS (Biological Industries, Israel). WM3314 cells (12.5 × 10^5^, generously provided by Carmit Levi, Tel-Aviv University), in the presence or absence of KLK4_S207A,L185D_ protein, were incubated in 200 µL of FBS-free DMEM, added to the pre-coated ThinCert cell culture inserts, and incubated for 18 h at 37 °C and 5% CO_2_. Invasive cells were stained with a DippKwik stain kit (American MasterTech Scientific) and detected by an EVOS FL Cell Imaging System (Life Technologies, Carlsbad, CA, USA), at ×4 magnification. The experiment was performed in triplicate, and the images were taken from the center field of each insert. Analysis of digitized images was performed using ImageJ software and a Cell Colony Edge Analyser.

### ERK phosphorylation assay

The experiment was performed using the WM3682 cell line as previously described with minor modifications^54^. The cells were grown in complete DMEM (Biological Industries) containing 10% FBS, penicillin, streptomycin, and l-glutamine until they reached 80% confluence. Cells were scraped with a cell scraper, diluted 1:2, and grown overnight in complete DMEM. Thereafter, 2.5 × 10^5^ cells were transferred into a 12-well plate with complete DMEM for 24 h. At this point, the medium was replaced with a starvation medium (FBS-free complete DMEM) for 18 h. After starvation, the cells were washed with PBS and incubated in a starvation medium containing 10 nM KLK4_WT_ and 0, 10, 100 and 1000 nM KLK4_S207A,L185D_ in a humidified incubator at 37 °C and 5% CO_2_ for 15 min. The positive control contained 10 nM KLK4_WT_ alone, and the negative control was incubated in starvation medium without any added protein. The cells were transferred to ice, and a lysis buffer [deoxycholate 0.5%, 25 nM NaF, 10 mM Na_2_PO_4_, 1 mM sodium orthovanadate, 5 mM EDTA (pH 7.4), 5 mM EGTA (pH 7.4), 100 mM NaCl, 2% Triton X-100, 2% *p*-nitrophenyl phosphate (PNPP) and a protease inhibitor cocktail (A2S, Israel)] was added. The cells were detached with a cell scraper, collected, incubated on ice for 10 min, and centrifuged at 14,000 g for 30 min, and the supernatants were transferred to fresh tubes. Western blot analysis was performed for all samples using anti-MAP kinase (Erk1/2) produced in rabbit or anti-MAP kinase, activated (diphosphorylated Erk1/2) antibody produced in mouse as a primary antibody (Sigma, MA, USA). A secondary HRP-linked anti-rabbit or anti-mouse antibody was then added, and the signal was developed using the EZ-ECL kit (Biological Industries). The chemiluminescent signal was imaged with Fusion FX (Vilber Lourmat, Germany). Equal amounts of total protein from cell lysate samples were loaded in each lane on the SDS-PAGE, as measured by a colorimetric BCA protein assay kit (Pierce Biotechnology, Rockford, IL, USA). The experiment was performed in triplicate, and a representative membrane image was selected for presentation.

### Cell viability assay

The effects of KLK4_S207A,L185D_ on the growth and survival of WM3682 and MCF7 cells were assessed by an XTT assay (2,3-bis [2-methoxy-4-nitro-5-sulfophenyl]-2H-tetrazolium-5-carboxanilide inner salt assay; Biological Industries). WM3682 or MCF7 cells were seeded (7500 cells per well) in a 96-well microplate (R&D Systems, MN, USA) and incubated at 37 °C at 5% CO_2_ for 24 h. The medium was then replaced with fresh DMEM supplemented with 10% FBS, penicillin, streptomycin, and l-glutamine, and the cells were incubated for 24 h at 37 °C at 5% CO_2_ in the presence or absence of 1 μM KLK4_S207A,L185D_. Viable cells from each condition were measured by reading the absorbance at 450 nm (UV), using a plate reader, as described in the manufacturer’s protocol. The UV readings of the cell-only control were normalized to 100%, and readings from cells treated with KLK4_S207A,L185D_ were presented as a percentage of the control.

### Statistical analysis

Data were analyzed with GraphPad Prism version 5.00 for Windows (La Jolla, CA). Data shown in all the figures are the means of triplicates from independent experiments, and error bars represent the standard error of the mean. Statistical significance was determined by column statistics and one-way ANOVA analysis. A *p* value < 0.05 was considered statistically significant.

## Supplementary Information

Supplementary Information.
