# Supplementary material for: A KLK4 proteinase substrate capture approach to antagonize PAR1
Source: Sci Rep. 2021 Aug 9;11:16170. doi: 10.1038/s41598-021-95666-4 (PMC8352894; doi:10.1038/s41598-021-95666-4)
Supplement: Supplementary file 1 — Supplementary Information. [file 41598_2021_95666_MOESM1_ESM.pdf]

Supplementary Materials

A KLK4 proteinase substrate capture approach to antagonize PAR1

Eitan Rabinovitch[1], Koishiro Mihara[2], Amiram Sananes[1], Marianna Zaretsky[3], Michael Heyne[1,4], Julia Shifman[4], Amir Aharoni[3], Morley D. Hollenberg[2] & Niv Papo[1*]

[1]Avram and Stella Goldstein-Goren Department of Biotechnology Engineering and the National Institute of Biotechnology in the Negev, Ben-Gurion University of the Negev, Beer-Sheva, Israel.

[2]Department of Physiology and Pharmacology, Cumming School of Medicine, University of Calgary, Calgary, Canada.

[3]Department of Life Sciences and National Institute of Biotechnology in the Negev, Ben-Gurion University of the Negev, Beer-Sheva, Israel.

[4]Department of Biological Chemistry, The Hebrew University of Jerusalem, Givat Ram Campus, Jerusalem 91906, Israel.

[*]To whom correspondence should be addressed. E-mail: papo@bgu.ac.il. Pre-publication correspondence should be sent to Niv Papo, Department of Biotechnology Engineering and the National Institute of Biotechnology, Ben-Gurion University of the Negev; P.O.B. 653, Beer-Sheva 84105, Israel; Phone: +972-50-2029729; email: papo@bgu.ac.il.

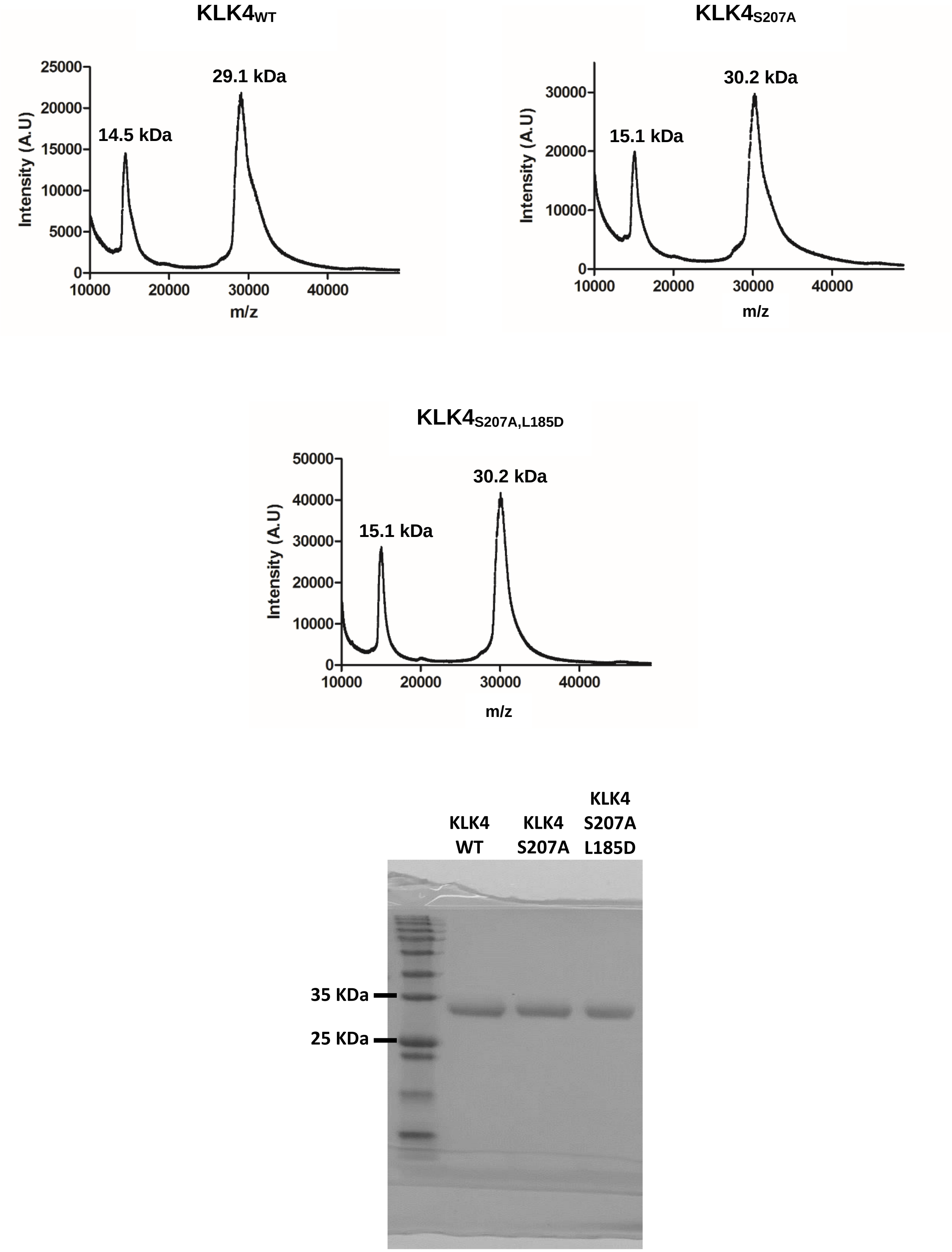

Figure S1. Mass spectrometry analysis for non-activated KLK4$_{WT}$ (29,111Da), KLK4$_{S207A}$ (30,213Da) and KLK4$_{S207A,L185D}$ (30,235Da). The lower panel includes a full length SDS-PAGE of KLK4$_{WT}$ and of KLK4$_{S207A}$ and KLK4$_{S207A,L185D}$ as selected purified pro-KLK4 variants.

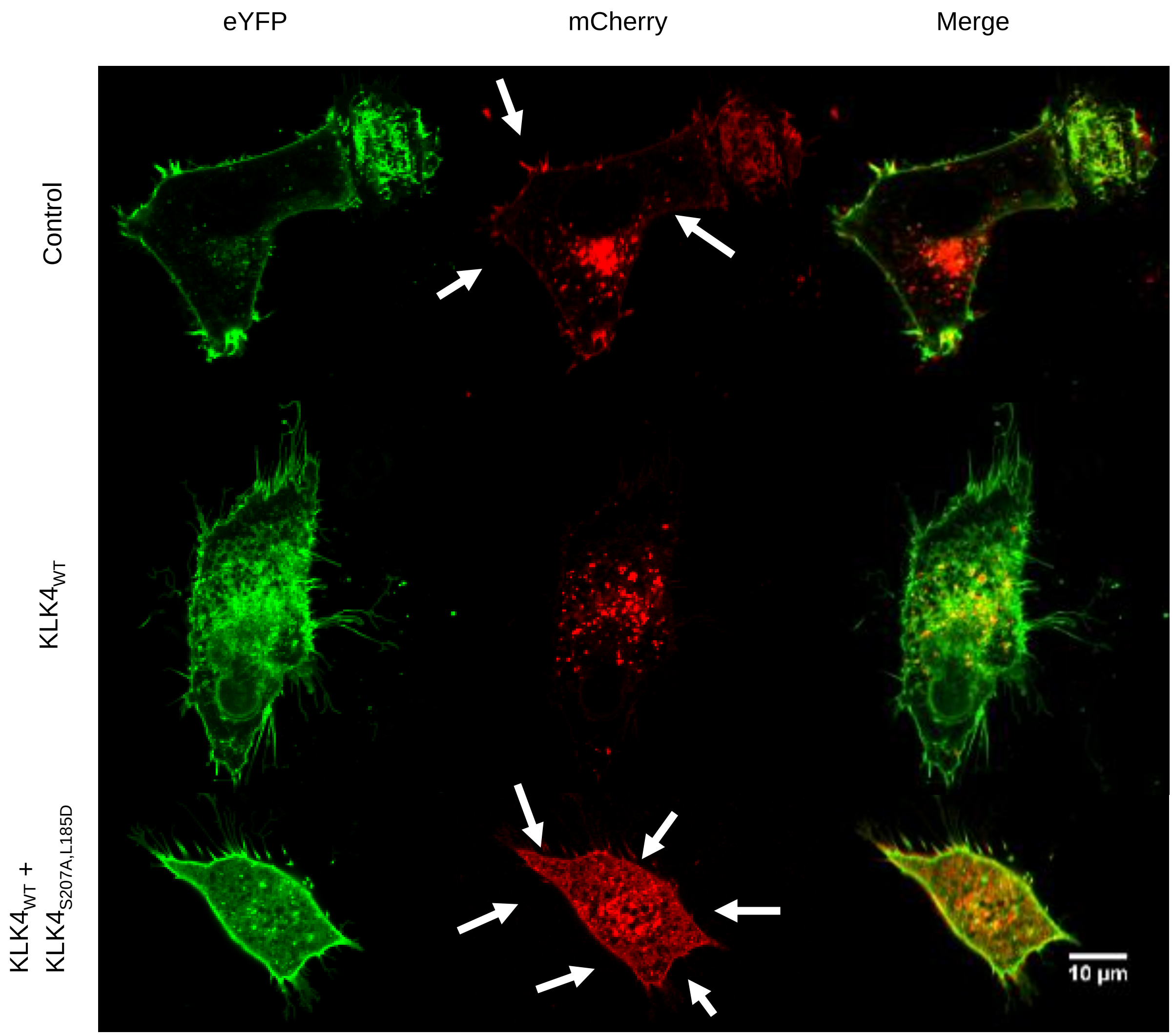

Figure S2. Confocal microscopy images of WM3682 cells transiently overexpressing an mCherry-PAR1-YFP construct and treated with PBS, 10 nM $KLK4_{WT}$, or a combination of 10 nM $KLK4_{WT}$ and 100 nM $KLK4_{S207A,L185D}$ for 1 h prior to analysis. Arrows point to visible membrane mCherry signals. A 20-µm scale bar is also shown.

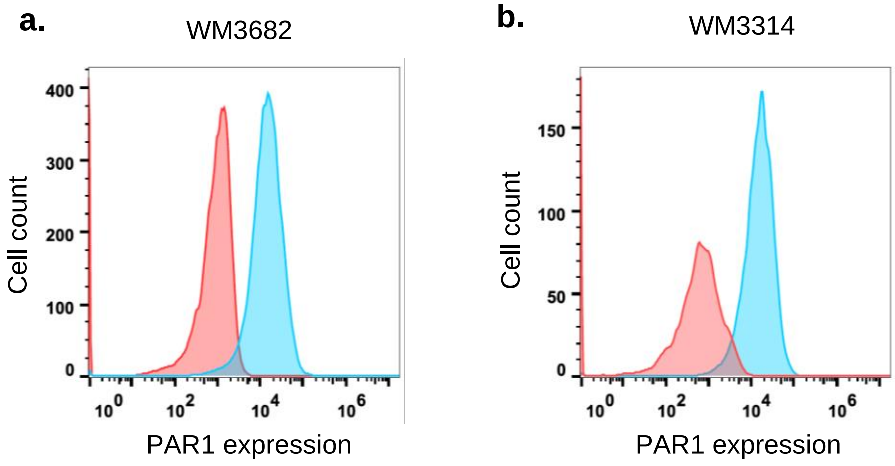

Figure S3. Flow-cytometry results for endogenous PAR1 expression in a) WM3682 and b) WM3314 cell lines. PAR1 was labeled with anti PAR1 APC-conjugated antibody, and the fluorescence intensity signals of unlabeled cells and labeled cells are represented by red and blue histograms, respectively.

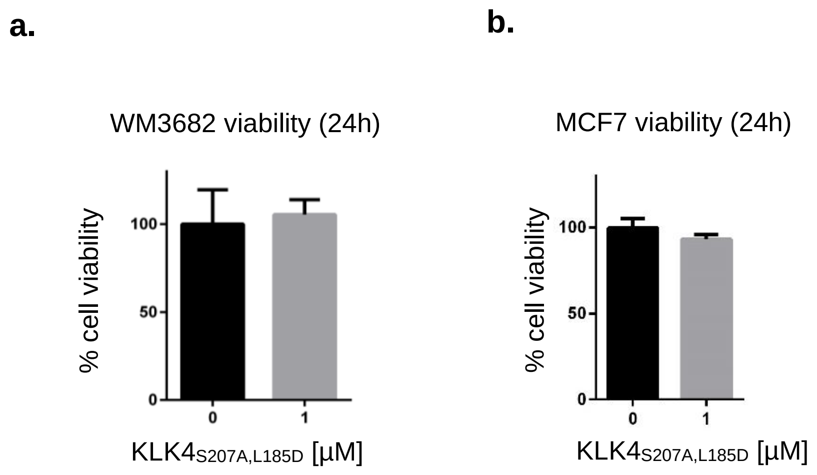

Figure S4. XTT viability assay for a) WM3682 and b) MCF7 cell lines upon incubation (for 24 h) in the presence or absence of 1 μM $KLK4_{S207A,L185D}$. The XTT assay is based on the cleavage of the tetrazolium salt XTT in the presence of an electron-coupling reagent to produce a soluble formazan salt. This conversion occurs only in viable cells.

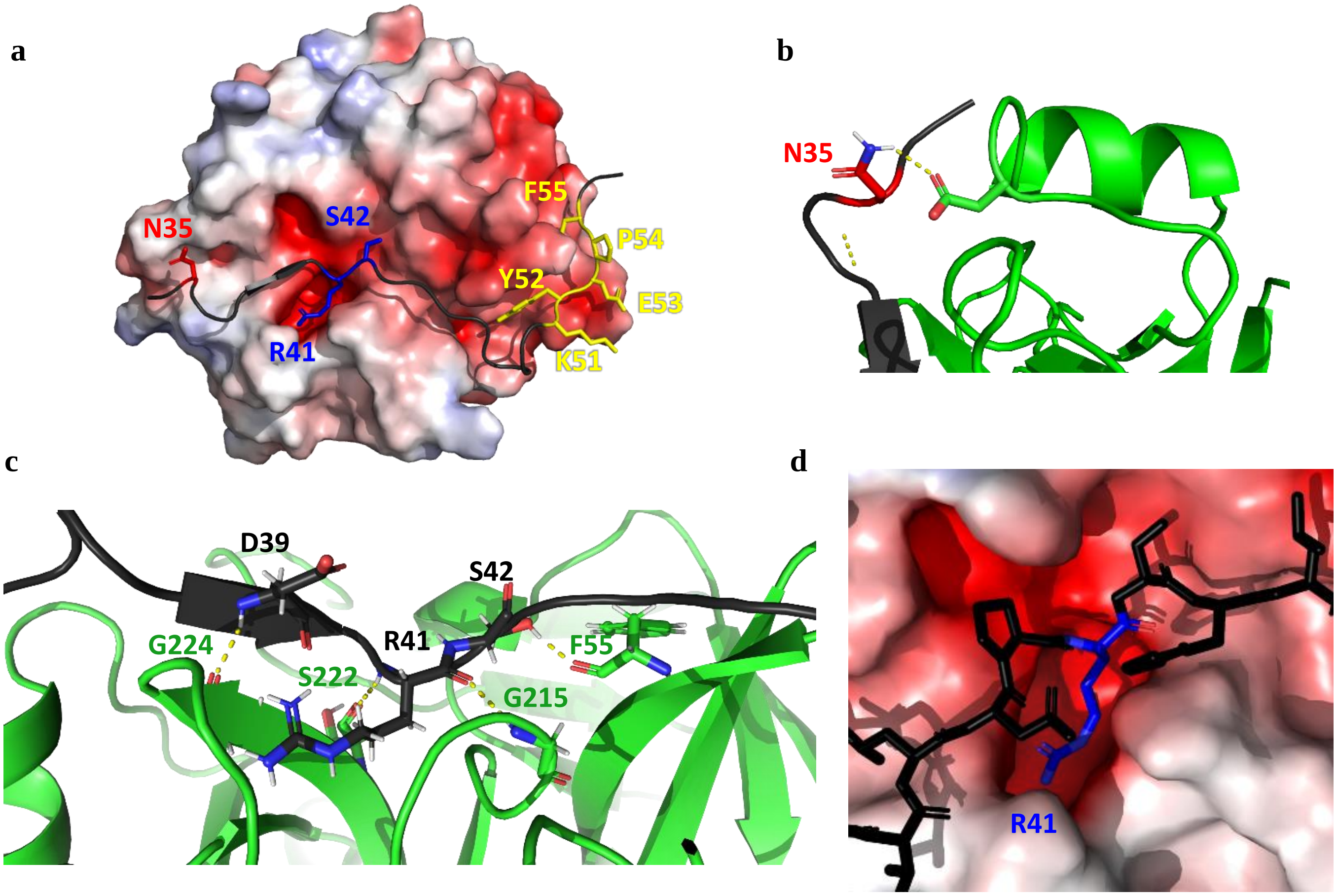

Figure S5. A model of $KLK4_{S207A,L185D}$ bound to the PAR1 peptide. (a) General representation of the three predicted binding sites between KLK4 (shown in a surface charge representation with a negative net charge colored in red, positive net charge colored in blue, and a neutral charge colored in white) and the PAR1 peptide (represented as a cartoon and sticks). The peptide may be divided according its three interaction sites with KLK4: 1) between the "hirudin-like binding domain" residues K51-F55 in the PAR1 peptide, colored in yellow, and the negatively charged surface cavity of KLK4; 2) between residue N35 in the PAR1 peptide (colored in red), which is predicted to form a hydrogen bond with residue D185 in KLK4, as shown in panel (b); and 3) between the cleavage site of the PAR1 peptide (residues R41-S42, colored in blue) and the catalytic pocket of KLK4, as shown in panels (c) and (d). KLK4 is shown in green, and the PAR1 peptide is shown in black. Hydrogen bonds are shown as yellow dashed lines. The positively charged residue R41 (in blue) of the PAR1 peptide buried within the negatively charged pocket of KLK4 is shown in red.

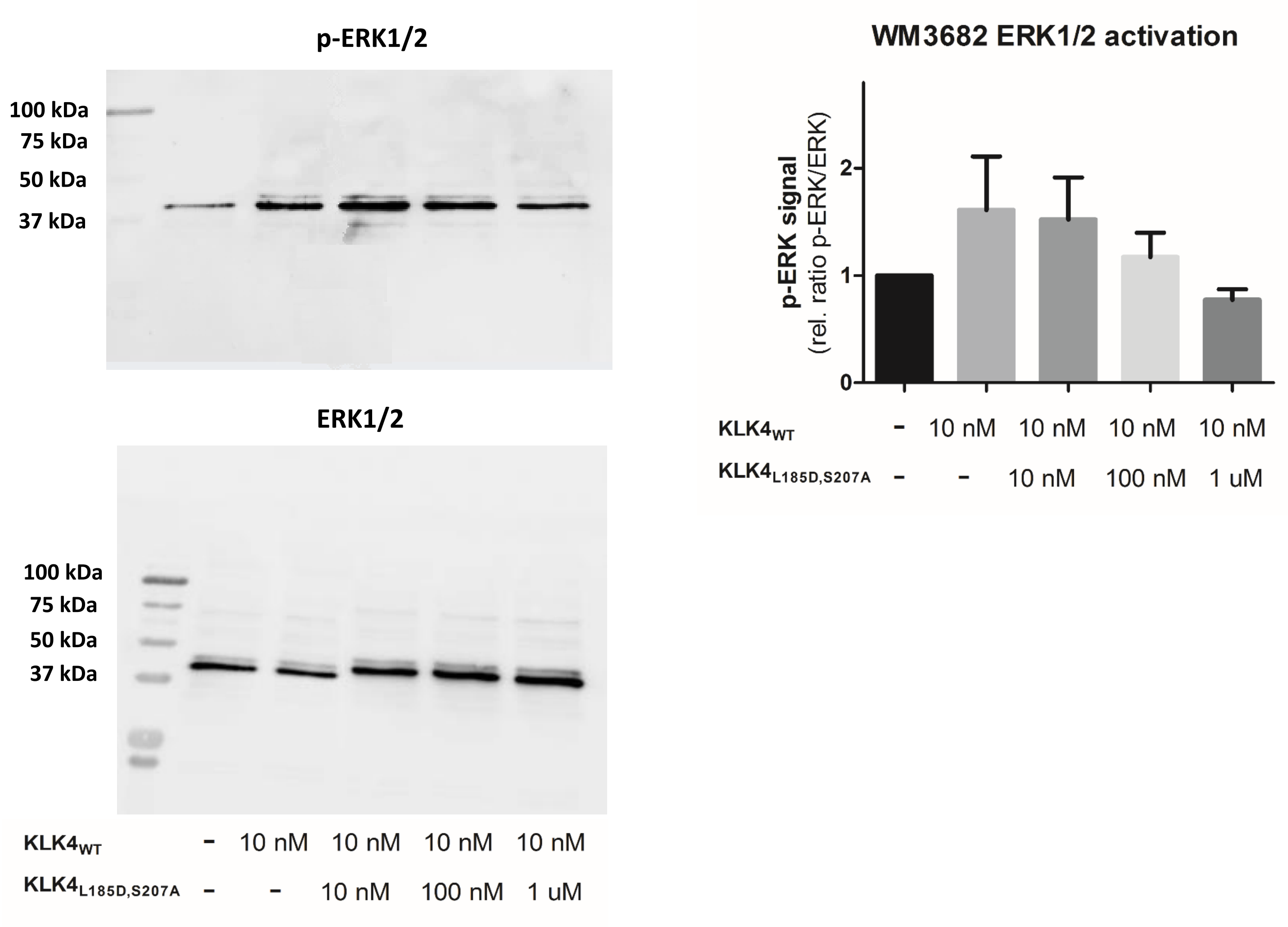

Figure S6. Effect of KLK4$_{L185D,S207A}$ on ERK 1/2 phosphorylation. WM3682 cells were treated for 15 min with PBS, 10 nM KLK4$_{WT}$, or with a mixture of 10 nM KLK4$_{WT}$ and 10 nM, 100 nM or 1 µM KLK4$_{L185D,S207A}$. Cells were lysed, and the cell lysates were subjected to western blot analysis. The ERK1/2 phosphorylation levels were calculated by dividing pERK1/2 band signal levels by ERK1/2 and β-actin band signal levels. For each sample, the phosphorylation band intensity was normalized to the ERK1/2 phosphorylation of untreated WM3682 cells (in which no KLK4$_{WT}$ and KLK4$_{L185D,S207A}$ were present). The highest phosphorylation level was obtained with KLK4$_{WT}$. The left panels show a full-length western blot gel for analysis of ERK and p-ERK levels.

Table S1. KLK4 site-directed mutagenesis and sequencing primers

Primer number	Name	Sequence
1	AOX Fwd	5'-GACTGGTTCCAATTGACAAGC
2	AOX Rev	5'-GGCAAATGGCATTCTGACAT
3	K4 S207A Fwd	5'-CATGTAATGGTGATGCAGGTGGTCCATTAATATGC
4	K4 S207A Rev	5'- GCATATTAATGGACCACCTGCATCACCATTACATG
5	K4 E98I Fwd	5'-GGTTCTCAAATGGTAATCGCATCTTTGTCAGTT
6	K4 E98I Rev	5'-AACTGACAAAGATGCGATTACCATTTGAGAACC
7	K4 E98Y Fwd	5'-GGTTCTCAAATGGTATACGCATCTTTGTCAGTT
8	K4 E98Y Rev	5'-AACTGACAAAGATGCGTATACCATTTGAGAACC
9	K4 E98F Fwd	5'-GGTTCTCAAATGGTATTCGCATCTTTGTCAGTT
10	K4 E98F Rev	5'-AACTGACAAAGATGCGAATACCATTTGAGAACC
11	K4 L185S Fwd	5'-ATTGTATGACCCAGAGTACCACCCTTCCATG
12	K4 L185S Rev	5'-CATGGAAGGGTGGTACTCTGGGTCATACAAT
13	K4 L185Q Fwd	5'-ATTGTATGACCCACAGTACCACCCTTCCATG
14	K4 L185Q Rev	5'-CATGGAAGGGTGGTACTGTGGGTCATACAAT
15	K4 L185N Fwd	5'-ATTGTATGACCCAAACTACCACCCTTCCATG
16	K4 L185N Rev	5'-CATGGAAGGGTGGTAGTTTGGGTCATACAAT
17	K4 L185H Fwd	5'-ATTGTATGACCCACACTACCACCCTTCCATG
18	K4 L185H Rev	5'-CATGGAAGGGTGGTAGTGTGGGTCATACAAT
19	K4 L185R Fwd	5'-ATTGTATGACCCAAGATACCACCCTTCCATG
20	K4 L185R Rev	5'-CATGGAAGGGTGGTATCTTGGGTCATACAAT
21	K4 L185K Fwd	5'-ATTGTATGACCCAAAGTACCACCCTTCCATG
22	K4 L185K Rev	5'-CATGGAAGGGTGGTACTTTGGGTCATACAAT
23	K4 L185E Fwd	5'-ATTGTATGACCCAGAGTACCACCCTTCCATG
24	K4 L185E Rev	5'-CATGGAAGGGTGGTACTCTGGGTCATACAAT
25	K4 L185D Fwd	5'-ATTGTATGACCCAGACTACCACCCTTCCATG
26	K4 L185D Rev	5'-CATGGAAGGGTGGTAGTCTGGGTCATACAAT
